# The Secretome of *Phanerochaete chrysosporium* and *Trametes versicolor* Grown in Microcrystalline Cellulose and Use of the Enzymes for Hydrolysis of Lignocellulosic Materials

**DOI:** 10.3389/fbioe.2020.00826

**Published:** 2020-07-17

**Authors:** Angela S. Machado, Fernanda Valadares, Tatiane F. Silva, Adriane M. F. Milagres, Fernando Segato, André Ferraz

**Affiliations:** Departamento de Biotecnologia, Escola de Engenharia de Lorena, Universidade de São Paulo, Lorena, Brazil

**Keywords:** basidiomycetes, biorefinery, cellobiohydrolases, glucoside hydrolases, secretome, sugarcane, white-rot fungi

## Abstract

The ability of white-rot fungi to degrade polysaccharides in lignified plant cell walls makes them a suitable reservoir for CAZyme prospects. However, to date, CAZymes from these species are barely studied, which limits their use in the set of choices for biomass conversion in modern biorefineries. The current work joined secretome studies of two representative white-rot fungi, *Phanerochaete chrysosporium* and *Trametes versicolor*, with expression analysis of cellobiohydrolase (CBH) genes, and use of the secretomes to evaluate enzymatic conversion of simple and complex sugarcane-derived substrates. Avicel was used to induce secretion of high levels of CBHs in the extracellular medium. A total of 56 and 58 proteins were identified in cultures of *P. chrysosporium* and *T. versicolor*, respectively, with 78–86% of these proteins corresponding to plant cell wall degrading enzymes (cellulolytic, hemicellulolytic, pectinolytic, esterase, and auxiliary activity). CBHI predominated among the plant cell wall degrading enzymes, corresponding to 47 and 34% of the detected proteins in *P. chrysosporium* and *T. versicolor*, respectively, which confirms that Avicel is an efficient CBH inducer in white-rot fungi. The induction by Avicel of genes encoding CBHs (*cel*) was supported by high expression levels of *cel7D* and *cel7*C in *P. chrysosporium* and *T. versicolor*, respectively. Both white-rot fungi secretomes enabled hydrolysis experiments at 10 FPU/g substrate, despite the varied proportions of CBHs and other enzymes present in each case. When low recalcitrance sugarcane pith was used as a substrate, *P. chrysosporium* and *T. versicolor* secretomes performed similarly to Cellic^®^ CTec2. However, the white-rot fungi secretomes were less efficient than Cellic^®^ CTec2 during hydrolysis of more recalcitrant substrates, such as acid or alkaline sulfite-pretreated sugarcane bagasse, likely because Cellic^®^ CTec2 contains an excess of CBHs compared with the white-rot fungi secretomes. General comparison of the white-rot fungi secretomes highlighted *T. versicolor* enzymes for providing high glucan conversions, even at lower proportion of CBHs, probably because the other enzymes present in this secretome and CBHs lacking carbohydrate-binding modules compensate for problems associated with unproductive binding to lignin.

## Introduction

Hydrolytic enzymes are major constituents of commercial enzymatic cocktails used in plant biomass conversion of pretreated lignocellulosic materials (Payne et al., 2015; Adsul et al., 2020). The cellulose and hemicellulose hydrolysis promoted by these enzymatic cocktails is the basis of modern biorefineries. However, improvement of the performance of these enzymatic cocktails is still necessary to advance cost effective production of plant biomass derivatives (Ellila et al., 2017; Adsul et al., 2020). To date, most of these enzymatic cocktails are developed for polysaccharide conversion to monosaccharides and contain diverse cellulolytic and hemicellulolytic enzymes, including glucoside hydrolases (GHs) and auxiliary activity enzymes, such as lytic polysaccharide monooxygenases (LPMOs), that cause oxidative cleavage of polysaccharides (Payne et al., 2015; Adsul et al., 2020). Both enzyme groups can contain carbohydrate-binding modules (CBMs) associated with the catalytic domain that enable efficient adsorption of the enzymes on insoluble polysaccharides, improving the enzymatic catalysis efficiency (Payne et al., 2015). Enzymatic accessibility is another critical parameter when plant biomass is used as a substrate, which usually makes pretreatment of the plant biomass material necessary before the enzymatic conversion processes (Chundawat et al., 2011). Residual lignin contained in pretreated materials can also cause unproductive binding of GHs and LPMOs, especially because CBMs also adsorb on lignin surfaces (Rahikainen et al., 2013; Siqueira et al., 2017; Santos et al., 2019).

Currently, ascomycetes are the main source for production of commercial enzymatic cocktails containing GHs and LPMOs destined for plant biomass conversion, because this class of fungi is amenable to genetic engineering, presents robust machinery to secrete such enzymes and is well developed from a bioprocess-engineering point of view (Juturu and Wu, 2014; Bischof et al., 2016; Druzhinina and Kubicek, 2017; Fitz et al., 2018). In ascomycete-based enzymatic cocktails, CBHs are the chief enzymes because they are uniquely able to cleave cellulose chains in a processive manner, releasing cellobiose with high efficiency (Payne et al., 2015). In contrast to ascomycetes, basidiomycetes involved in natural wood decay have been less developed as a source of such enzymes, despite the fact that this fungal class has evolved to degrade polysaccharides in lignified substrates and thus appears to be a logical reservoir for searching for proper plant biomass conversion enzymes (Floudas et al., 2012; Bentil et al., 2018; Valadares et al., 2019). In this context, sequencing of fungal genomes has increasingly opened an opportunity to search for new enzymes in wood decay fungi, which has resulted in the discovery of hitherto unexplored enzymes with particular characteristics that enable improved plant biomass conversion (Ohm et al., 2014; Lange et al., 2019).

Lignocellulosic substrates or pure polysaccharides and their degradation products have been used to induce secretion of plant biomass degrading proteins in fungi (Szabó et al., 1996; Suto and Tomita, 2001; Ahamed and Vermette, 2009; Juturu and Wu, 2014; Payne et al., 2015; Bischof et al., 2016; Presley and Schilling, 2017; Lin et al., 2019). Certain studies of white-rot fungi have shown that Avicel (a commercial preparation of microcrystalline cellulose) and its biodegradation products act as GH inducers in the model basidiomycete *Phanerochaete chrysosporium* (Uzcategui et al., 1991; Broda et al., 1995; Szabó et al., 1996; Suzuki et al., 2010). Other white-rot species have been less explored as plant biomass degrading protein producers (Rytioja et al., 2014; Bentil et al., 2018; Valadares et al., 2019).

Available studies indicate that *P. chrysosporium* produce a more diverse group of CBHs than the model cellulase producer, the ascomycete *Hypocrea jecorina* (anamorph *Thrichoderma reesei*). Seven CBHs are recognized in *P. chrysosporium* genome (Cel7A-F/G and one Cel6), and five of them are efficiently secreted when the fungus is cultured in cellulose, cello-oligosaccharides or plant biomass substrates (Martinez et al., 2004; Ravalason et al., 2008; Suzuki et al., 2009; Adav et al., 2012). In contrast, *T. reesei* encodes and produces only one CBHI (Cel 7A) and one CBHII (Cel 6A) (Peterson and Nevalainen, 2012; Kunamneni et al., 2014).

Previous literature suggests that distinct enzymes, useful for plant biomass conversion, can be found in wood decay fungi. In this context, the current work explores CBH-enriched secretomes of two white-rot fungi, *P. chrysosporium* and *T. versicolor*. Expression analysis of *cbh* genes was performed via RT-qPCR assays, and the produced enzymes were deeply characterized and used for digestion of lignin-free polysaccharides and plant biomass substrates. Both white-rot fungi secretomes enabled efficient cellulose and hemicellulose conversion to monosaccharides.

## Materials and Methods

### Cultures of *P. chrysosporium* and *T. versicolor* in Microcrystalline Cellulose (Avicel) or Glucose as a Single Carbon Source

Stock cultures of *P. chrysosporium* (RP-78, ATCC MYA-4764) and *T. versicolor* (BAFC 266, Mycological Culture Collection of the Department of Biological Sciences, University of Buenos Aires, Argentina - Levin et al., 2007) were maintained at 4°C on 2.0% malt extract (Synth, SP, Brazil) and 0.2% yeast extract (Vetec, Rio de Janeiro, Brazil) agar slants containing a wood chip slice.

Both basidiomycetes were cultured on Norkrans medium (Eriksson and Johnsrud, 1983) containing 20 g/L Avicel (Fluka, PH101) or 20 g/L glucose as a single carbon source. Cultures were grown in 1 L Erlenmeyer flasks containing 300 mL medium and inoculated with 10^4^ spores/L in the case of *P. chrysosporium* or 500 mg/L of blended mycelium (Machado and Ferraz, 2017) in the case of *T. versicolor*. Cultures were maintained at 37°C for *P. chrysosporium* and 27°C for *T. versicolor* under 150 rpm rotary shaking for up to 12 days. At least three replicate culture flasks were inoculated for each culture period.

After defined culture periods, flask contents were filtered through Miracloth^®^ (Millipore Sigma, Burlington, MA, United States). Solids representing fungal mycelium (from glucose cultures) or fungal mycelium plus residual Avicel were frozen in liquid nitrogen and stored at −80°C. Liquid broths from at least three replicate cultures were combined and further filtered through 0.45 μm polyethersulfone membranes (Sartorius Stedim, Göttingen, Germany). Filtrated broths were then concentrated via ultrafiltration through 10 kDa cut-off polyethersulfone membranes (Biomax Millipore, Janfrey, NH, United States) up to 35 times, depending on the fungal species and culture period (see Supplementary Figure S1 for details). Samples of the concentrated culture broths were assayed for filter paper activity (FPA) and total proteins. The remaining concentrated broths were freeze-dried and stored at −18°C.

### Protein Determination and Enzymatic Assays

Concentrated culture broths or dissolved freeze-dried broths were assayed for total proteins and for filter paper activity (FPA) as described in Ghose (1987). CBH (EC 3.2.1.176 and EC 3.2.1.91, for reducing and non-reducing end activity, respectively) (Wood and Bhat, 1988), endoglucanase (EC 3.2.1.4) (Ghose, 1987), endoxylanase (EC 3.2.1.8) (Bailey et al., 1992), β-glucosidase (EC 3.2.1.21) and β-xylosidase (EC EC 3.2.1.37) (Tan et al., 1987) were determined according to the indicated methods. At least three independent cultures from each fungal species were combined before concentration and freeze-drying of culture broths. After dissolution, freeze-dried broths were assayed in three analytical determinations of each enzymatic activity. Standard deviation for analytical triplicates varied less than 5% of the reported value.

### SDS-PAGE and Proteomic Analysis

Freeze-dried culture broths were dissolved, and 30 μg of total protein from each sample was assessed via SDS-PAGE using a Mini Protean Tetra Cell System (Bio-Rad, Hercules, CA, United States). Protein bands were stained with 0.2% (w/v) Coomassie Brilliant Blue G 250, 50% (v/v) ethanol, and 10% (v/v) acetic acid. Each lane from the SDS-PAGE gels was divided into five fragments to cover the entire range of protein molar masses. Gel fragments were stored in a 1:1 methanol/water solution containing 0.1% formic acid and analyzed according to the protocols developed by the Life Sciences Core Facility (LaCTAD) at University of Campinas, SP, Brazil^1^. Gel-fragments were prepared by incubation with 100 μL of 100 mM ammonium bicarbonate/acetonitrile (1:1) for 30 min. After centrifugation, the liquid fraction was discarded, and solids were suspended in 500 μL acetonitrile until the gel became white. Decanted solids were dried and treated with 100 μL of 10 mM dithiothreitol (DTT) solution in 100 mM ammonium bicarbonate at 56°C for 30 min. Liquids were discarded, and dried solids were treated with 100 μL of 55 mM iodoacetoamide at room temperature for 30 min. Liquids were discarded again, and solids were digested with 100 μL of 13 mg/mL trypsin solution in 50 mM ammonium bicarbonate. Digestion with trypsin lasted overnight at 37°C. After centrifugation, the supernatant was collected and transferred to another microcentrifuge tube. Solids were further extracted for 15 min with 100 μL of 100 M ammonium bicarbonate/acetonitrile (1:1), and liquid fractions were combined for subsequent peptide analysis via LC-MS/MS.

For peptide analysis, 5 μL of digested sample was trapped in a Symmetry C18 precolumn (5 μm × 180 μm × 20 mm – Waters, Milford, MA, United States). The trapped sample was eluted inline onto an HSS T3 column (1.8 μm × 75 μm × 100 mm – Waters, Milford, MA, United States) using a solvent gradient ranging from 7% (v/v) to 85% (v/v) acetonitrile containing 0.1% (v/v) formic acid at 0.4 μL/min with a runtime of 73 min. LC-MS/MS data acquisition was achieved in a XEVO G2 Xs QToF mass spectrometer equipped with a nanolockspray source in the positive ion mode (Waters, Corp., Milford, MA. United States). Instrument calibration was performed with the MS/MS fragment ions of GFP [Glu 1]-Fibrinopeptide B with a doubly charged ion [M + 2H]^2+^ = 785,84206 *m*/*z* (Waters, Corp., Milford, MA, United States). Data-independent scanning (MSE) experiments were performed by switching between low (3 eV) and high (15–50 eV) collision energies applied to a trap “T-wave” cell filled with argon. A scan time of 0.5 s was used to acquire data from *m*/*z* 50 to 2000.

Data were processed with Progenesis QI 4.0 (Non-linear Dynamics). The processing parameters included an automatic mode for MS-TOF resolution and chromatographic peak width. The low-energy and high-energy detection thresholds were optimized by software. Protein identification was fitted to a minimum of three fragment ions matched per peptide, a minimum of five fragment ions matched per protein, a minimum of one unique peptide matched per protein, one possible trypsin missed cleavage, carbamidomethylation of cysteine as a fixed modification and oxidation of methionine as a variable modification, and a maximum false positive discovery rate (FDR) in auto mode. The FDR for peptide and protein identification was determined based on a search of a reversed database, which was generated automatically using the Progenesis QI 4.0 software. All protein hits were identified with a confidence of >95%. The databases (Filtered Models) for *P. chrysosporium* v2.2 (RP-78) and *T. versicolor* v1.0 (FP-101664 SS1) used in the protein search were obtained at the MycoCosm portal from DOE Joint Genome Institute (Floudas et al., 2012; Ohm et al., 2014). The number of sequences and the average protein length (in amino acids) for *P. chrysosporium* and *T. versicolor* were 13602/408 and 14530/422, respectively.

### Real Time qPCR Analysis

The primers used for amplification of the cDNA of *P. chrysosporium* genes for *cel7A-E, cel6*, and *actin* were the same reported by Suzuki et al. (2009). The sequences of primers used for amplification of the cDNA fragments derived from *T. versicolor cel7A-D* and *actin* were designed based on analysis of the non-coding regions of the genes (3′ UTRs) following the method established by Suzuki et al. (2009) (Supplementary Table S1). In the case of the *cel6* gene from *T. versicolor*, the primers were designed within the coding region of the gene (Supplementary Table S1). The corresponding transcripts of the *actin* gene were used as the internal control in both cases. Relative gene expression was calculated with the 2^–ΔΔ*CT*^ method using the same target gene expressed in mycelia recovered from catabolic-repressed cultures grown on 20 g/L glucose medium for 5 days as the calibrator sample (Livak and Schmittgen, 2001).

Frozen fungal mycelia were ground to a fine powder using a mortar and pestle in the presence of liquid nitrogen. Total RNA was extracted with an illustra^TM^ RNAspin Mini Isolation Kit (GE Healthcare, Chicago, IL, United States) and treated with an RNase-free DNase set (Promega, Madison, Wisconsin, United States) according to the manufacturer’s instructions. First-strand cDNA was synthesized using 800 ng treated RNA and 100 μM oligo (dT)_24_ primer SuperScript^TM^ III Reverse Transcriptase (Life Technologies^TM^, Thermo Fisher) according to the manufacturer’s instructions.

Real-time qPCR was performed in a 7500 Fast Real-Time PCR System (Life Technologies^TM^, Thermo Fischer). The reaction wells contained 2.5 μL of prepared cDNA (diluted 25 times), 5 μL of Maxima SYBR Green/Rox qPCR Master Mixes buffer (Life Technologies^TM^, Thermo Fischer), 0.4 μL of forward primer (100 mM), 0.4 μL of reverse primer (100 mM), and 11.7 μL of H_2_O. The reaction conditions were 50°C for 2 min, 95°C for 10 min and 40 repetitions of 95°C for 15 s and 60°C for 1 min. All reactions were subjected to the same analysis conditions, and the results were normalized to the signal of the passive reference dye ROX to correct for fluctuations in the reading resulting from variations in volume and evaporation throughout the reaction. Two biological replicates were assayed in three technical replicates per cDNA sample.

### Sequence Analysis and Comparison of CBHI Proteins

The sequences of the CBHI from *P. chrysosporium* (JGI 137372, JGI 2971601, JGI 3024803, and JGI 2976245) and *T. versicolor* (JGI 112163, JGI 110790, and JGI 124366) identified in the secretomes were checked against the InterPro 77.0 database^2^ to validate the domains and used for multiple alignments with Geneious v4.8.5 using CBHI from *T. reesei* QM6a as a model (JGI 123989). Tridimensional modeling of *T. versicolor* CBHI-Cel7C (JGI 112163) was performed using the server I-TASSER (Yang and Zhang, 2015). Cel7A from *Heterobasidium irregulare* (formely *H. annosum*)^3^ (Momeni et al., 2013) was used as a search model owing to 78% similarity between both CBH sequences. The CBHs from *T. reesei* (Cel7A) and *P. chrysosporium* (Cel7D) were used to compare structural differences (PDB id 1CEL and 1GPI, respectively). The figures were generated using PyMOL Molecular Graphics System version 2.3 (Schrödinger, LLC).

### Enzymatic Hydrolysis of Lignocellulosic Substrates

Three different sugarcane-derived substrates were prepared as described in previous studies and selected to represent lignified plant biomass samples with varying recalcitrance to enzymatic digestion. The selected materials corresponded to (A) the pith region of mature sugarcane stalks of a low recalcitrant hybrid denominated H89 (Costa et al., 2013, 2016); (B) alkaline-sulfite pretreated sugarcane bagasse prepared as described in Reinoso et al. (2018); and (C) dilute acid pretreated sugarcane bagasse prepared as described in Santos et al. (2018), using a 90 min reaction time at 150°C. Chemical composition of these substrates was determined according to Ferraz et al. (2000).

Pretreated materials were digested inside 2 mL microcentrifuge tubes containing 20 mg of milled sugarcane biomass sample (passing 0.84 mm screen) suspended in a 1 mL reaction volume. Reaction solutions contained the enzymatic preparations at 10 FPU/g substrate dissolved in 50 mM sodium acetate (pH 4.8) containing 0.01% sodium azide. Reaction tubes were agitated at 120 rpm and 45°C for periods up to 72 h and sampled for monomeric sugar analysis according to Valadares et al. (2016).

## Results

### Secretomes of *P. chrysosporium* and *T. versicolor* Grown in Microcrystalline Cellulose

**FIGURE 1:**
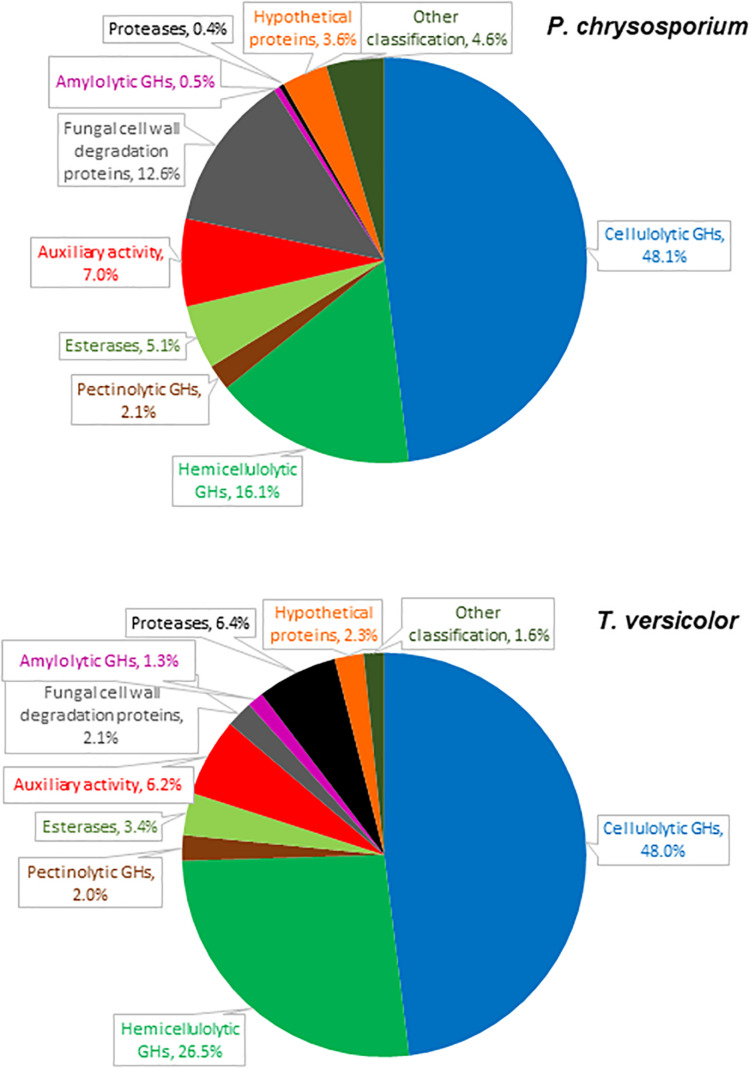
Relative abundance of proteins detected in cultures of *P. chrysosporium* and *T. versicolor* grown for 12 days in 20 g/L Avicel as sole carbon source.

Secretomes produced by *P. chrysosporium* and *T. versicolor* grown in microcrystalline cellulose (Avicel) were characterized and used for plant biomass saccharification. Avicel was used as the sole carbon source to enrich the secretomes with CBHs (Tangnu et al., 1981; Szabó et al., 1996; Payne et al., 2015). Increasing FPA levels were detected with extended culture of both white-rot fungi, with *P. chrysosporium* providing FPA (242 FPU/L) four times higher than *T. versicolor* (59 FPU/L) at the longest culture period (12 days) (Supplementary Figure S1). The secretomes contained several cellulolytic and hemicellulolytic activities (Table 1), resulting from a broad protein diversity, which was revealed by LC-MS/MS (Figure 1 and Supplementary Tables S2, S3). Comparison of the two white-rot fungi showed that *P. chrysosporium* provided the highest FPA, CBH and endoglucanase activities (Table 1). In contrast, *T. versicolor* showed significantly higher titers for β-glucosidase and for the xylanolytic complex. The reference cellulolytic cocktail, Cellic^®^ Ctec2 (Cannella et al., 2012; Bischof et al., 2016), differs from white-rot secretomes because it contains higher CBH and β-glucosidase activities, which consistently result in higher FPA (Table 1).

**TABLE 1:** Enzymatic activities determined from freeze-dried culture extracts prepared from *P. chrysosporium* and *T. versicolor* grown in 20 g/L Avicel as sole carbon source for 12 days.

Fungi	Enzymatic activity* in the freeze-dried culture extracts (IU/mg protein)					
	FPA	CBH	EG	β-gluc	endo-Xyl	β-Xyl
*P. chrysosporium*	0.36	0.42	22.0	2.1	180	3.0
*T. versicolor*	0.13	0.03	11.8	20.0	894	9.6
Cellic^®^ Ctec2**	0.95	1.90	25	46.0	96	0.5

*FPA, filter paper activity measured with paper strips; CBH, cellobiohydrolases measured with Avicel substrate; EG, endoglucanases measured with carboxymethyl cellulose substrate; β-gluc, β-glucosidases measured with pNPG substrate; endoXyl, endoxylanases measured with birchwood xylan substrate; and β-xyl, β-xylosidases measured with pNPX substrate. **Cellic^®^ Ctec2 is a commercial enzyme blend acquired from Sigma (SAE0020). At least three independent cultures from each fungal species were combined before concentration and freeze-drying of culture broths. After dissolution, freeze-dried broths were assayed in three analytical determinations of each enzymatic activity. Standard deviation for analytical triplicates varied less than 5% of the reported value.

The *P. chrysosporium* and *T. versicolor* secretomes contained 56 and 58 detectable proteins, respectively (Supplementary Tables S2, S3). Most of the identified proteins presented signal peptides (Petersen et al., 2011) or signals for non-classical secretion pathways (Bendtsen et al., 2004). Only one protein in the *P. chrysosporium* and four in the *T. versicolor* secretomes were intracellular. Plant cell wall degrading enzymes (cellulolytic, hemicellulolytic, pectinolytic, esterase, and auxiliary activity) predominated among the detected proteins, altogether representing 78 and 86% of the peptides detected by LC-MS/MS in the *P. chrysosporium* and *T. versicolor* secretomes, respectively (Figure 1). Minor proteins included fungal cell wall degradation proteins, amylolytic enzymes, proteases, hypothetical proteins, and proteins with other classifications.

**FIGURE 2:**
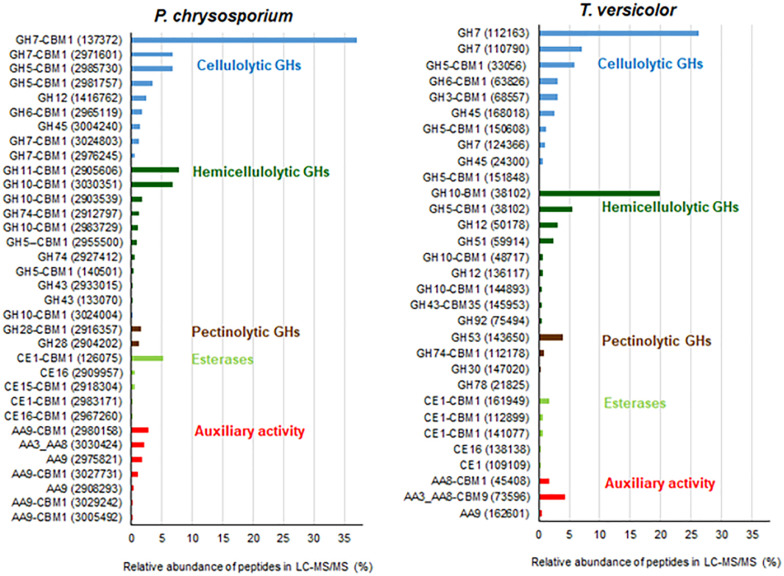
Relative abundance of plant cell wall degrading proteins detected in cultures of *P. chrysosporium* and *T. versicolor* grown for 12 days in 20 g/L Avicel as sole carbon source. JGI identification protein numbers are indicated in parenthesis. Relative abundance is expressed considering the total plant cell wall degrading proteins as 100%. Original relative abundance of plant cell wall degrading proteins in each secretome was 78 and 86% for *P. chrysosporium* and *T. versicolor*, respectively.

Figure 2 highlights the relative abundance of each plant cell wall degrading protein in both secretomes. GH7-CBHIs corresponded to 47 and 34% of the total plant cell wall degrading proteins in *P. chrysosporium* and *T. versicolor*, respectively. GH6-CBHIIs were also detected in both fungi, with 1–3% relative abundance. The predominance of CBHs in both secretomes confirms that cellulose and its degradation products are useful CBH inducers in white-rot fungi (Broda et al., 1995; Szabó et al., 1996; Suzuki et al., 2010). The second most abundant enzyme group in both fungi was GH10 and GH11 endoxylanases in *P. chrysosporium* (17%) and GH10 endoxylanases in *T. versicolor* (21%). Endoglucanases appeared in the sequence, with GH5, GH12 and GH45 families in *P. chrysosporium* (14%) and GH5 and GH45 families in *T. versicolor* (10.5%) (Figure 2). One GH3 β-glucosidase was detected in the *T. versicolor* cultures (3.1%), but putative β-glucosidases were not detectable in the *P. chrysosporium* secretome. Several auxiliary activity (AA) enzymes were also detected, with 6 LPMOs (EC 1.14.99.54-C1-hydroxylating and EC 1.14.99.56-C4-dehydrogenating) from AA9 (4%) and one cellobiose-dehydrogenase (CDH-EC 1.1.99.18) from AA3_AA8 (2%) in *P. chrysosporium*. In contrast, only one putative LPMO from AA9 (0.6%) but one CDH from AA3_AA8 (4.7%) and one cytochrome c domain from AA8 appended to a CBM1 (1.7%) were found in *T. versicolor*. Accessory enzymes involved in cleavage of hemicellulose side groups, including GHs and carbohydrate esterases (CEs), as well as putative pectinolytic GHs, also appeared in both fungal secretomes (Figure 2).

CBHs are major proteins in the commercial enzymatic cocktails prepared for efficient plant biomass saccharification (Table 1) because they are able to decompose long cellulose chains into cellobiose via a processive mode of action (Payne et al., 2015). CBHs also formed the main enzymes in the secretomes of both white-rot fungi cultured in 20 g/L Avicel as the sole carbon source. However, the processivity of each particular CBH and the velocity at which CBHs cleave glucosidic bonds depend on the enzyme structure (Momeni et al., 2013; Taylor et al., 2018) and on the substrate characteristics (Kurasin and Väljamäe, 2011). In this context, the type of CBH present in each white-rot fungi secretome was detailed to reveal CBH variations among the well-known *P. chrysosporium* (Munoz et al., 2001; von Ossowski et al., 2003; Kurasin and Väljamäe, 2011; Tachioka et al., 2017; Taylor et al., 2018) and the understudied *T. versicolor*.

**FIGURE 3:**
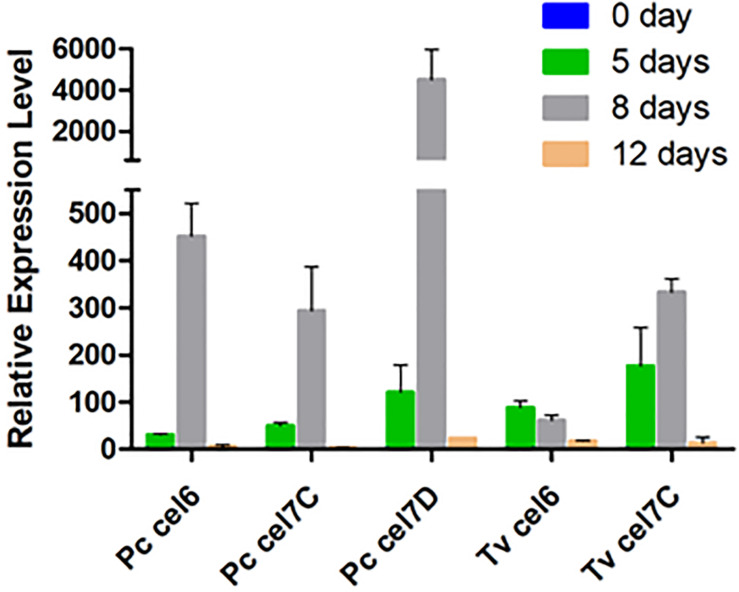
Relative transcription levels of genes encoding cellobiohydrolase in *P. chrysosporium* (*Pc*) and *T. versicolor* (*Tv*) grown in 20 g/L Avicel as sole carbon source. Actin was used as house-keeping gene in both cases. Relative transcription levels are reported as 2^–ΔΔ*Ct*^ using catabolic repressed culture (mycelia recovered from 20 g/L glucose for 5 days) as the calibrator sample. Two biological replicates were assayed in analytical triplicate for qPCR.

Among CBHs, CBHI-Cel7D (JGI 137372) predominated in the secretome of *P. chrysosporium* (37%), followed by CBHI-Cel7C (JGI 2971601) (6.8%), CBHII (JGI2965119) (1.7%), CBHI-Cel7E (JGI 3024803) (1.3%), and CBHI-Cel7F/G (JGI 2976245/JGI 2976248) (0.5%). In *T. versicolor* cultures, CBHI-Cel7C (JGI 112163) predominated (26%) over CBHI-Cel7B (JGI 110790) (7.0%), followed by CBHII (JGI 63826) (3.1%), and CBHI-Cel7D (JGI 124266) (1.0%). Real-time qPCR provided the pattern of *cbh* gene transcripts in the fungal mycelia grown during the cultivation periods. For both fungal species, *cbh* transcripts measured at 5, 8, and 12 days attained maximal levels at day 8, decreasing afterward (Figure 3). This behavior suggested an initial induction period as already demonstrated for *P. chrysosporium* K-3 strain (Suzuki et al., 2009), whereas the late decrease in *cbh* transcripts is likely associated with repression caused by glucose released from Avicel at advanced culture periods (Daly et al., 2019). However, total FPA progressively accumulated in the culture media along the 12 days culture period (Supplementary Figure S1), indicating a delay between gene transcription and enzyme secretion and extracellular accumulation (Midorikawa et al., 2018; Lin et al., 2019).

The CBHs detected in the secretome dovetail with the *cbh* gene transcript levels, since CBHI-Cel7D (JGI 137372) and CBHI-Cel7C (JGI 112163) predominated in the *P. chrysosporium* and *T. versicolor* secretomes, respectively (Figure 2), and the corresponding transcripts (*Pc cel7D* and *Tv cel7C*) prevailed in the studied mycelium of each species (Figure 3). Detection of *Pc cel6* and *Pc cel7C*, and *Tv cel6* (Figure 3) also agreed with the occurrence of CBHII (JGI2965119) and CBHI Cel 7C (JGI 2971601) in the *P. chrysosporium* and CBHII (JGI 63826) in the *T. versicolor* secretome, respectively (Figure 2). The primers used for amplification of *cel7A* and *cel7B* fragments from cDNA of *P. chrysosporium* confirmed that the genes were not being transcribed under the tested conditions, consistent with the absence of the corresponding enzymes in the secretome of this fungal species. *Pc cel7E* transcripts were only barely detectable in *P. chrysosporium* (data not shown), as was the case for CBHI-Cel7E (JGI 3024803). In *T. versicolor* cultures, *cel7A* transcripts and the corresponding protein were not detectable. However, in contrast with previous observations, CBHI-Cel7B (JGI 110790) and CBHII (JGI 63826) appeared in the *T. versicolor* secretome, whereas the primers used for amplification of *cel7B* and *cel6* fragments from cDNA (Table S1) were applied but did not amplify any fragments.

**FIGURE 4:**
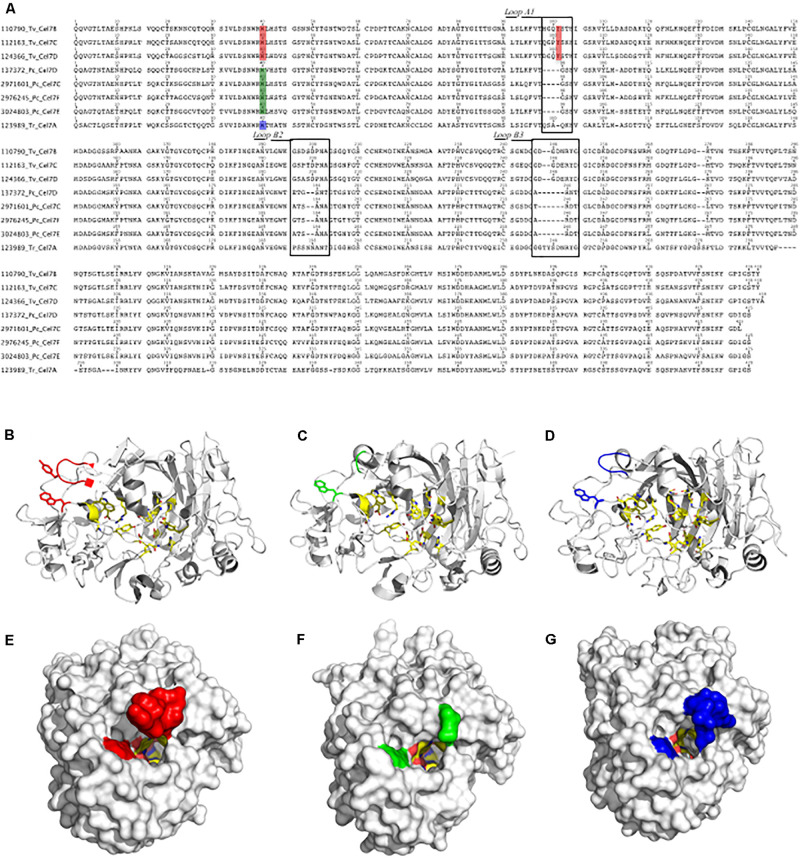
Sequence alignment of CBHIs from *T. versicolor* (110790 Tv Cel7B, 112163 Tv Cel7C and 124366 Tv Cel7D), *P. chrysosporium* (137372 Pc Cel7D, 2971601 Pc Cel7C, 2976245 Pc Cel7F, and 3024803 Pc Cel7E) and *T. reesei* (123989 Tr Cel7A) **(A)**. Structural models for CBHI 112163 Tv Cel7C **(B,E)**, CBHI 137372 Pc Cel7D **(C,F)** and 123989 Tr Cel7A **(D,G)**. In **(A)** the squares indicate the loop A1, loop B2, and loop B3. Amino acids in the tunnel entrance are also highlighted in red, green and blue **(A)**, which can be compared at the structures **(B–D)** or in the surface **(E–G)** in the corresponding colors.

CBHs were further compared through a sequence analysis that highlighted certain structural differences among the CBHs from *P. chrysosporium* and *T. versicolor* detected in the secretome. CBHI from *T. reesei* (JGI 123989) was used as a reference (Figure 4A). Alignment of the catalytic modules and molecular modeling showed several common structures, such as a conserved cellulose-biding site and the catalytic triad (Glu-214, Asp-216 and Glu-209) (Figure 4A) (Momeni et al., 2013). However, the whole sequences confirmed that CBHIs from *T. versicolor* lack the typical CBM from family 1 found in *P. chrysosporium* and *T. reesei* (data not shown).

Structural analysis showed that the catalytic cores of CBHI were typical, with a β-sandwich surrounded by loops (Figures 4B–D). Compared to CBHIs from *P. chrysosporium* and *T. reesei*, the Loop A1 in CBHIs from *T. versicolor* differs in length and sequence and contains a tyrosine at position 101 (Y-101 in Figures 4A,B). The tunnel entrance in *T. versicolor* CBHIs is narrower than that in *P. chrysosporium* and *T. reesei* CBHIs (Figures 4E–G). Other differences among these CBHIs occur in Loop B2 (Figure 4A), which lacks two amino acid residues in *P. chrysosporium* CBHIs and is substituted in *T. versicolor* compared to the *T. reesei* enzyme that contain two asparagine at positions 197 and 198. Finally, in loop B3 (Figure 4A), CBHIs from *T. versicolor* and *P. chrysosporium* are shorter by two and six residues, respectively, compared to *T. reesei* CBHI.

### Saccharification of Sugarcane Substrates by the Crude Secretomes of White-Rot Fungi

**FIGURE 5:**
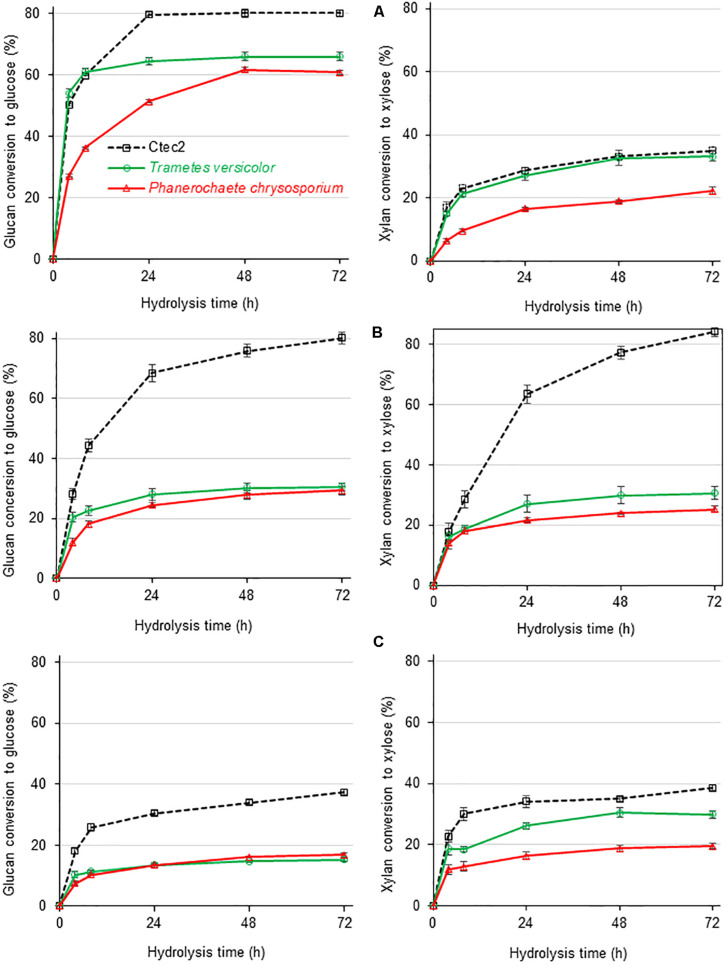
Glucan (left) and xylan (right) conversion during enzymatic hydrolysis of three different sugarcane-derived substrates by secretomes from white-rot fungi as compared to Cellic^®^ Ctec2 at a fixed load of 10 FPU/g of substrate but varied compositional enzymatic activities as reported in Table 1. **(A)** Sugar cane pith, **(B)** alkaline-sulfite pretreated sugarcane bagasse, and **(C)** dilute acid pretreated sugarcane bagasse, substrates. At least five independent cultures from each fungal species were combined before concentration and freeze-drying of culture broths. After dissolution, freeze-dried broths were used for plant biomass saccharification in three analytical determinations.

Three different sugarcane-derived substrates were digested with the secretomes produced by *P. chrysosporium* and *T. versicolor* with the aim of evaluating their performance on more complex, plant biomass-derived substrates (Figure 5). For all experiments, the protein loads were adjusted to provide 10 FPU/g substrate. Cellic^®^ CTec2 served as an upmost reference enzymatic cocktail in the same experiments. As can be depicted from Table 1 data, the protein load and enzymatic activities in the digestion tubes varied considerably to assure an equal load of 10 FPU/g substrate in all cases.

The *T. versicolor* secretome, with low FPA and CBH activities, required a high protein load (75 mg protein/g substrate) to reach 10 FPU/g substrate. The *P. chrysosporium* secretome presented more balanced CBH:EG:β-glucosidase levels, providing 10 FPU/g substrate with 28 mg protein/g substrate, whereas Cellic^®^ CTec2, presenting a proper CBH:EG:β-glucosidase balance, required minimal protein loads (10 mg protein/substrate) to reach the same 10 FPU/g substrate. In addition to traditional cellulases, Cellic^®^ CTec2 also contains a sufficient number of xylanases and LPMOs (Cannella et al., 2012), enhancing the digestion efficiency for complex plant biomass substrates. Finally, owing to the low FPA and CBH and high endoxylanase and β-xylosidase activities, experiments with the *T. versicolor* secretome occurred with a disproportionally high xylanase load, corresponding to 27000 IU/g substrate compared to 1000 and 500 IU/g substrate for Cellic^®^ Ctec2 and the *P. chrysosporium* secretome, respectively.

The three enzymatic cocktails were used for saccharification of diverse sugarcane-derived substrates: (A) sugarcane pith that does not need pretreatment (Costa et al., 2013, 2016); (B) alkaline-sulfite pretreated material (Reinoso et al., 2018); and (C) dilute acid pretreated material (Santos et al., 2018). The chemical compositions of these substrates are presented in Supplementary Table S4. The major characteristics of these substrates indicate that sugarcane pith is a non-pretreated plant biomass sample rich in cellulose and β-1-3/β-1-4 mixed linkage glucan, presenting a low crystallinity index, and a low lignin content (Costa et al., 2016). The xylan contained in sugarcane pith retains its original structure, presenting a high number of side groups, such as acetylated groups (Costa et al., 2016). Alkaline-sulfite pretreated sugarcane bagasse is depleted in lignin (approximately 50% of lignin is removed during pretreatment), has almost all cellulose preserved and its xylan lacks major acetyl and feruloyl side groups owing to saponification during alkaline pretreatment (Reinoso et al., 2018). Dilute acid pretreated sugarcane bagasse is enriched in crystalline cellulose and lignin because almost all amorphous cellulose and 90% of xylan are removed during pretreatment (Santos et al., 2018).

Based on the glucan conversion levels provided by the reference Cellic^®^ Ctec2 cocktail, the three substrates presented decreasing digestibility, with sugarcane pith ≥ alkaline sulfite >> dilute sulfuric acid (Figure 5). For pretreated substrates, the high CBH titers detected in Cellic^®^ Ctec2 (Table 1) seem essential for high glucan conversion to glucose, since the reference cocktail performed significantly better than the *P. chrysosporium* and *T. versicolor* secretomes (Figure 5). In contrast, for low recalcitrance materials, such as the sugarcane pith (Costa et al., 2016), glucan conversion provided by both white-rot secretomes reached efficiency values comparable to those observed during saccharification with Cellic^®^ Ctec2 (Figure 5A).

During sugarcane pith digestion, the xylan conversion levels were significantly lower than the glucan conversion levels for all three enzymatic cocktails (Figure 5). Selected sugarcane hybrids contain a pith region comprising a very low recalcitrant lignocellulosic material rich in parenchyma cells that do not require pretreatment for efficient enzymatic glucan conversion to glucose (Costa et al., 2013; Costa et al., 2016). However, the absence of pretreatment results in preserved xylan structures containing all side group decorations, which restrain efficient xylan hydrolysis (Varnai et al., 2014).

### Saccharification of Sugarcane-Derived Substrates by the Secretomes of White-Rot Fungi Supplemented With β-Glucosidases

**FIGURE 6:**
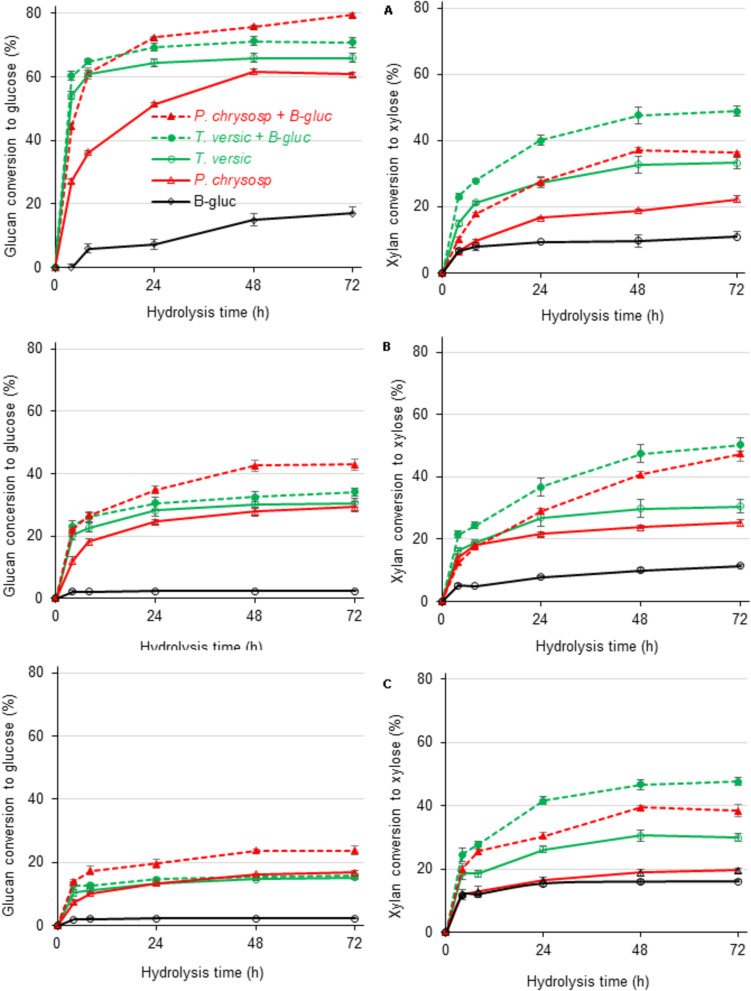
Glucan (left) and xylan (right) conversion during enzymatic hydrolysis of three different sugarcane-derived substrates by concentrate secretomes from white-rot fungi additional 10 IU of β-glucosidase/g of substrates (labeled with + B-glic). **(A)** Sugar cane pith, **(B)** alkaline sulfite pretreated sugarcane bagasse, and **(C)** dilute acid pretreated sugarcane bagasse substrates. At least five independent cultures from each fungal species were combined before concentration and freeze-drying of culture broths. After dissolution, freeze-dried broths were used for plant biomass saccharification in three analytical determinations.

The lack of β-glucosidase in the *P. chrysosporium* secretome was experimentally overcome by supplementation with commercial *Aspergillus niger* β-glucosidase, which also presents some xylanase activity (Valadares et al., 2016; Figure 6). Supplementation with β-glucosidase enhanced the glucan conversion levels of all substrates. With β-glucosidase supplementation, the *P. chrysosporium* secretome provided higher glucan and xylan conversion than the *T. versicolor* secretome. However, in sugarcane pith, supplementation with *A. niger* β-glucosidase presented a simple additive effect, since glucan conversion to glucose was enhanced 19% in the experiment with *P. chrysosporium* secretome compared to the 17% provided by *A. niger* β-glucosidase alone (Figure 6A). The *T. versicolor* secretome was less reliant on β-glucosidase supplementation, probably because it presented a high original β-glucosidase activity (Table 1). For the pretreated substrates, supplementation with β-glucosidase provided synergic action for glucan hydrolysis, because the enhanced glucan conversion surpassed the values provided by *A. niger* β-glucosidase alone (Figures 6B,C). A remarkable enhancement in xylan hydrolysis efficiency was also noted when the white-rot secretomes were supplemented with *A. niger* β-glucosidase, probably associated with the high xylanase activity described for this commercial preparation (Valadares et al., 2016). The enhanced xylan hydrolysis could also enhance glucan hydrolysis owing to progressive xylan removal from cellulose fibrils during the hydrolysis process (Varnai et al., 2014).

## Discussion

The enzymatic activities measured in the secretome of *P. chrysosporium* and *T. versicolor* (Table 1) and the relative abundance of proteins detected by LC-MS/MS (Figure 2) were in good agreement. For example, xylanase activity (endo-xylanase and β-xylosidase) was higher in *T. versicolor* than in *P. chrysosporium* cultures (Table 1), which agrees with the higher relative abundance of hemicellulolytic enzymes in the secretome of *T. versicolor*. The high β-glucosidase activity in *T. versicolor* cultures and the near absence of this activity in *P. chrysosporium* cultures also agrees with the detection of significant GH3 β-glucosidase in the secretome of *T. versicolor* but the absence of putative β-glucosidases in the *P. chrysosporium* secretome. Actually, *P. chrysosporium* has intracellular β-glucosidases and proper mechanisms for cellobiose transportation through the cell membrane instead of extracellular β-glucosidases (Tsukada et al., 2006). Endoglucanases also appeared at higher relative abundance in *P. chrysosporium* (14%) than in *T. versicolor* (10.5%), consistent with the higher endoglucanase activity in the *P. chrysosporium* secretome (Table 1). In contrast to previous remarks, *T. versicolor* presented significantly lower activity on crystalline cellulose than *P. chrysosporium* (Table 1), although its secretome presented relatively abundant CBHs (37.5%) compared to the *P. chrysosporium* secretome (48.7%).

The lack of CBMs in *T. versicolor* CBHs seems critical in explaining the low activity of the *T. versicolor* secretome on crystalline cellulose. In contrast, in addition to higher CBH abundance, the *P. chrysosporium* secretome contained abundant LPMOs and CDH (Figure 2 and Supplementary Tables S2, S3) that could boost hydrolysis of crystalline cellulose. FPA followed a trend similar to that of CBH activity, since the activities were significantly higher in *P. chrysosporium* than in *T. versicolor* (Table 1).

Further comparison of the main CBHs detected in both fungal species (CBHI-Cel7D – JGI 137372 in *P. chrysosporium* and CBHI-Cel7C – JGI 112163 in *T. versicolor*) with CBHI from *T. reesei* indicated that the basidiomycete enzymes present a more open cellulose-binding tunnel, which is related to the shorter loops (Figure 4). Previous studies based on computational analysis demonstrated that enzymes with shorter loops dissociate faster from substrate, showing a low inhibition rate by cellobiose and a high capacity to degrade microcrystalline cellulose because these enzymes can attach to the substrate with an endo-initiation mode of action (Kurasin and Väljamäe, 2011). In addition, the CBHIs from *T. versicolor* contain a tyrosine in the tunnel entrance, which in combination with tryptophan (W40 and Y101 highlighted in red, Figures 4A, B, and E) provides an extra binding platform for the enzyme, helping to drive the cellulose chain end into the catalytic site tunnel; this structure was already demonstrated in a similar CBHI produced by the basidiomycete *Heterobasidion irregulare* (Momeni et al., 2013).

Regarding plant biomass substrates, the hydrolytic performance of the three enzymatic cocktails varied considerably according to the characteristics of the substrate and the polysaccharide under evaluation (Figure 5). This varied performance primarily reflects the diverse composition of enzymes in each cocktail, although the same 10 FPU/g of substrate was maintained in all cases.

The *T. versicolor* secretome provided higher glucan conversion levels than the *P. chrysosporium* secretome, despite presenting a lower CBH proportion in the enzymatic cocktail (Table 1 and Figure 2). The higher efficiency of the *T. versicolor* secretome was especially evidenced during hydrolysis of sugarcane pith (Figure 5A). In this substrate, the initial glucan hydrolysis rate (up to 8 h of reaction time) provided by the *T. versicolor* secretome reached the same values observed for Cellic^®^ Ctec2. It is noteworthy that sugarcane pith presents high amounts of β-1-3/β-1-4 mixed linkage glucans and a low crystallinity index (Costa et al., 2016). Therefore, the very high β-glucosidase activity and sufficient endoglucanases in the *T. versicolor* secretome (Table 1) likely compensate for the lower proportion of CBH in its secretome. Excess β-glucosidase has also been reported to help in diminishing unproductive CBH adsorption on lignin, since β-glucosidase strongly adsorbs to this component (Jung et al., 2020).

The higher xylan conversion levels provided by the high xylanase activity in the *T. versicolor* secretome (similar to that provided by Cellic^®^ Ctec 2 – Figure 5A) could also facilitate the access of cellulolytic enzymes to cellulose in complex substrates (Varnai et al., 2014), favoring glucan conversion by this secretome. Therefore, the high endoxylanase and β-xylosidase titers detected in the *T. versicolor* secretome (Table 1) and the great diversity of xylan degrading enzymes in its secretome, including acetyl-xylan esterases (Supplementary Table S3 and Figure 3), seems to have favored significant xylan hydrolysis in sugarcane pith (Figure 5A). However, compared with the reference cocktail Cellic^®^ Ctec2, the *T. versicolor* secretome failed to promote the same efficiency in xylan hydrolysis in alkaline-sulfite pretreated substrate (Figure 5B). This substrate is more recalcitrant than sugarcane pith and presents xylan structures that lack side decorations, especially acetyl groups, which are mostly removed by the alkaline pretreatment (Reinoso et al., 2018).

Another factor associated with the more efficient glucan conversion by *T. versicolor* than *P. chrysosporium* can be attributed to lack of CBMs in CBHsI (Figure 2 and Supplementary Table S3). The lack of CBM in CBHsI from *T. versicolor* seems to be a limitation for hydrolysis of pure crystalline cellulose (Table 1) but not for digestion of lignified substrates, since less unproductive binding on lignin occurs with this type of CBH (Strobel et al., 2016; Lu et al., 2017). Indeed, some reports emphasize conflicting effects of CBMs in CBHs during hydrolysis of lignified substrates because they favor CBH attachment to cellulose chains, favoring cellulose hydrolysis efficiency; however, CBMs also adsorb unproductively to lignin, decreasing the amount of available CBH for cellulose processing (Strobel et al., 2016; Zhang et al., 2016; Lu et al., 2017).

In summary, the comparison of the white-rot fungi secretomes acting on lignified (Figure 5) versus lignin-free substrates (Table 1) suggests that *P. chrysosporium* secretome (with higher FPA and CBH activities) is more efficient to digest crystalline cellulose present in lignin-free substrates such as filter paper and Avicel because *P. chrysosporium* secretome presents higher amounts of CBHs, which contain CBMs. However, when both secretomes were applied to lignified substrates, at fixed 10 FPU/g substrate, *T. versicolor* provided higher glucan conversions, even presenting a lower proportion of CBHs, probably because the other enzymes present in this secretome and CBHs lacking CBMs compensate for problems associated with unproductive binding to lignin. Therefore, future exploring of *T. versicolor* CBHs seems relevant for preparation of new enzymatic cocktails used for plant biomass saccharification.

## Conclusion

Integrated studies of secretome, CBH transcription and enzymatic hydrolysis of varied substrates highlighted that microcrystalline cellulose (Avicel) was useful in inducing abundant CBHs in cultures of *P. chrysosporium* and *T. versicolor* white-rot fungi, enabling production of fungal secretomes for capable digestion of complex-lignified substrates. CBHI-Cel7D-CBM1 (JGI 137372) predominated in the *P. chrysosporium* secretome, whereas CBHI-Cel7C lacking CBM (JGI 112163) was the more abundant enzyme in the *T. versicolor* secretome. On a fixed 10 FPU/g substrate, these secretomes performed similarly to a commercial enzymatic cocktail (Cellic^®^ Ctec 2) when acting on a low-recalcitrant sugarcane pith substrate. However, efficient digestion of alkali or acid pretreated sugarcane-derived substrates required higher CBH proportion in the enzymatic cocktail, detected only in Cellic^®^ Ctec2. Comparison of the two white-rot fungi secretomes indicated that *T. versicolor* performed more efficiently in lignified substrates, even at a relatively low CBH proportion, likely helped by its high abundance of GH3 β-glucosidase and the presence of enzymes from the xylanolytic complex, including GH10 endoxylanases and CE1 acetylxylan esterases. *T. versicolor* CBHIs also lacked CBMs, which could have contributed to lower unproductive binding of these CBHs to the lignin contained in the complex sugarcane-derived substrates. Therefore, *T. versicolor* enzymes induced by Avicel seem to be good candidates for future research on preparation of new enzymatic cocktails used for hydrolysis of lignified substrates.

## Data Availability Statement

All datasets generated for this study are included in the article/Supplementary Material.

## Author Contributions

AM performed most of the experiments, the data interpretation, and discussion. FV, TS, and AMM participated in secretome and enzyme expression studies, the data interpretation, and discussion. FS participated in secretome and enzyme expression studies, protein modeling, data interpretation, and discussion. AF conceived the study, participated in data interpretation, discussion, and prepared the manuscript. All authors read and approved the final manuscript.

## Conflict of Interest

The authors declare that the research was conducted in the absence of any commercial or financial relationships that could be construed as a potential conflict of interest.

## Footnotes

1 https://www.lactad.unicamp.br/en/proteomics

2 http://www.ebi.ac.uk/interpro/

3 https://www.ncbi.nlm.nih.gov/pubmed/?term=23303184
