# Supplementary material for: The Secretome of Phanerochaete chrysosporium and Trametes versicolor Grown in Microcrystalline Cellulose and Use of the Enzymes for Hydrolysis of Lignocellulosic Materials
Source: Front Bioeng Biotechnol. 2020 Jul 17;8:826. doi: 10.3389/fbioe.2020.00826 (PMC7379840; doi:10.3389/fbioe.2020.00826)
Supplement: Supplementary file 1 [file Data_Sheet_1.pdf]

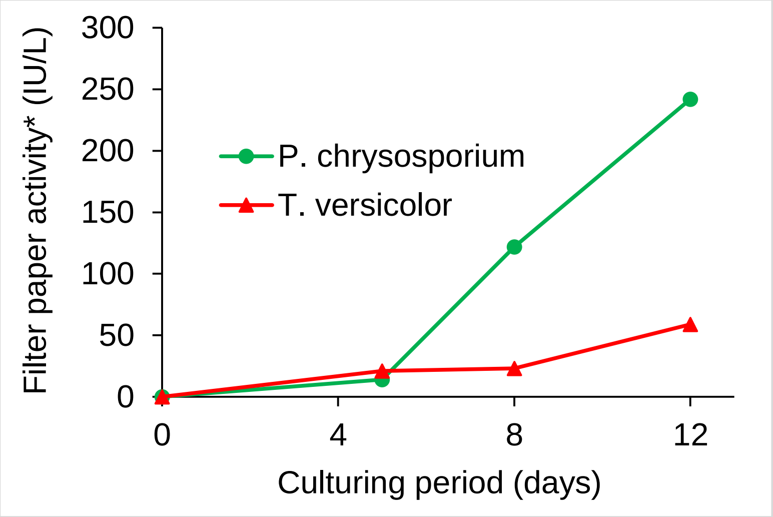

Figure S1. Filter paper activity detected in secretomes of *P. chrysosporium* and *T. versicolor* grown in 20 g/L Avicel as sole carbon source. (*) Reported filter paper activities were determined in concentrate culture extracts and properly recalculated to the original predicted activity in non-concentrate secretomes. At least three independent cultures from each fungal species were combined before concentration. Concentration rates of the culture broths depended on the fungal species and culturing period as folow. *P. chrysosporium*: 30, 25, and 17 times for culturing periods of 5, 8 and 10 days, respectively; *T. versicolor*: 35, 25, and 20 times for culturing periods of 5, 8 and 10 days, respectively. FPA determination is based on multiple dilutions of the assayed culture broth as reported in Ghose et al. (1987).

Table S1. Primers used for amplification of the cDNA of *Trametes versicolor*

Gene	(5'→3') Sequence	Size(pb)
Tv_*cel6*_Fwd	GGTTCTCCTACGACCGTCTC	109
Tv_*cel6*_Rev	AGCAGAGTAGTAGGGGCTCA	
Tv_*cel7A*_Fwd	CCGTCACCTTCTCGAACATCA	109
Tv_*cel7A*_Rev	AAGCGATGGGTGAGTGGAAG	
Tv_*cel7B*_Fwd	ACCGTCGTCTTCTCCAACAT	124
Tv_*cel7B*_Rev	GAGAGGAACATCTAGGGTGGC	
Tv_*cel7C*_Fwd	GAGAACAGCGAGGCGAACT	143
Tv_*cel7C*_Rev	CGGTATGTCTGCGATGATGGT	
Tv_*cel7D*_Fwd	AGTTCGGTCCTATCGGCTCT	104
Tv_*cel7D*_Rev	ACAGGCGTCTACACATCACC	
Tv_*actin*_Fwd	AGGTCAAGATCGTTGCTCCC	112
Tv_*actin*_Rev	CGTCGTACTCCTGCTTCGAG	

Table S4. Chemical composition of sugarcane substrates used in enzymatic digestion experiments

Sugarcane substrates	Chemical composition (%, w/w)				
	Glucan	Hemicellulose			Lignin
		Xylan	Arabinosyl	Acetyl	
Sugarcane pith	50.4±0.3	14.8±0.1	3.5±0.1	2.7±0.6	12.9±0.2
Alkaline-sulfite pretreated material	54.4±0.6	20.3±0.1	1.5±0.1	0.03±0.03	14.7±0.5
Dilute acid pretreated material	51.9±0.6	9.6±0.1	0.6±0.1	0.9±0.4	30.0±0.7
